# Structure and Electronic Properties of Transition Metal Doped Kaolinite Nanoclay

**DOI:** 10.1186/s11671-017-2188-4

**Published:** 2017-06-14

**Authors:** Liangjie Fu, Huaming Yang

**Affiliations:** 1 Centre for Mineral Materials, School of Minerals Processing and Bioengineering, Central South University, Changsha, 410083 China; 2 Peter A. Rock Thermochemistry Laboratory and NEAT ORU, University of California Davis, One Shields Avenue, Davis, CA 95616 USA; 3 Hunan Key Lab of Mineral Materials and Application, Central South University, Changsha, 410083 China

**Keywords:** Kaolinite nanoclay, Transition metal, Doping, Electronic structure, Dispersion-corrected density functional theory

## Abstract

In this work, a series of transition metal (Cr, Mn, Fe, and Co) doped kaolinite nanoclays were investigated by density functional theory (DFT) calculations. The influence of metal doping on geometric structure and electronic structure of kaolinite was analyzed. The ferromagnetic (FM), antiferromagnetic (AFM), and nonmagnetic (NM) states of transition metal (TM) doped kaolinite structures were studied. The crystal volume, lattice parameters, bond length, charge, and spin were calculated by dispersion-corrected density functional theory (DFT-D2). The results indicated that Cr^3+^ and Fe^3+^ dopants showed more stable under AFM state, while Mn^3+^ preferred both AFM and FM states, and Co^3+^ dopant preferred NM state. Also, the transition metal doping could induce lattice volume expansion and some dopant states in the band gap.

## Background

Kaolin-group nanoclay minerals, as a result of hydrothermal alteration and/or weathering processes, have unique physical properties because of their layered structure, small grain size, and most importantly the hydrated surface with plenty of hydroxyl groups. It has attracted the attention of researchers in materials chemistry, environmental chemistry, and mineral physics [1–11]. Kaolinite, one of the most abundant nanoclay minerals on Earth, has been wildly used in plastics, catalysis, and the cement industry. Further functionalization of kaolinite as novel support materials has attracted more and more attentions in various fields. Kaolinite can simply serve as support materials to mix with other nanoparticles to form phase change materials for solar energy utility [4, 5] or coated with doped oxide to form conductive powders for applications in conductive fields [9, 12]. The hybridization of kaolinite with functional nanoparticles was found to enhance the photocatalytic activity of Pd–ZnO and the luminescence properties of CdS through a synergistic effect [6, 7]. The surface properties of kaolinite were modified by anchoring some functional groups at the surface [13, 14] or by acid activation pretreatment for further improvement [2].

The structures and energetics of kaolin-group minerals have been extensively investigated experimentally [15–17] and theoretically [18–22]. Theoretical study of heavy metal adsorption on the kaolinite surface were studied for Cd, Cu, Hg, and Ni(II) adsorption [23], in which adsorption ability of kaolinite clay for ions were found in the order of Ni > Cu > Cd > Hg(II). The adsorption and diffusion of Pb(II) [24, 25] and uranyl [26] on the kaolinite (001) surface were studied [24–26], and the adsorption behavior in aqueous system was also reported later [27, 28]. The influence of Mg, Ca, and Fe doping on kaolinite surface, and the subsequent adsorption and penetration of H_2_O into the interlayer were studied [29]. The adsorption energies of H_2_O on doped kaolinites (001) were found less than undoped surface. The electronic structure of kaolinite with and without intrinsic defects has been studied by the standard density functional theory (DFT) functionals and hybrid functionals [30]. However, not until recently have the structure evolutions during the dehydroxylation, dealumination, and silica condensation process of kaolinite are modeled by DFT calculations [1, 31, 32]. The removal of Al in kaolin-group materials greatly altered the geometry and electronic properties of these layer materials and improved their support effect [1, 2].

Metal doping, as a well-known method to modify the structure and properties of compounds, has been theoretically studied for Al_2_O_3_ [33], TiO_2_ [34], MOF [35], and other solids [36]. To explore the changes in structure and properties of kaolinite nanoclay upon transition metal (TM) doping would be interesting for this layered clay material. In this work, a series of Cr, Mn, Fe, and Co doped kaolinite nanoclay were studied by DFT calculations and focused on the influence of metal doping on geometric structure and electronic structure of kaolinite nanoclay. The possible ferromagnetic (FM), antiferromagnetic (AFM), and nonmagnetic (NM) states of these transition metal doped kaolinite structures were studied. The lattice parameters, bond length, charge, and spin were optimized and calculated by dispersion-corrected density functional theory (DFT-D2).

## Methods

All calculations were performed with the program CASTEP (Cambridge Sequential Total Energy Package) code [37], based on first-principle DFT. Generalized gradient approximation (GGA) with the exchange-correlation potential by Perdew, Burke, and Ernzerhof (PBE) was used for the calculations [38]. Grimme’s DFT-D2 dispersion corrections were included to account for Van der Waals dispersion interactions [39]. An energy cutoff of 500 eV was applied using the ultrasoft pseudo-potential plane-wave formalism [40]. The Monkhorst–Pack [41] grid with 2 × 2 × 3 *k*-point mesh was used for geometrical relaxation and electronic structure calculations. The self-consistent total energy in the ground state was effectively obtained by the density-mixing scheme [42]. For the geometry optimizations, the convergence threshold for self-consistent field (SCF) tolerance was set to 1.0 × 10^−6^ eV/atom, all forces on the atoms were converged to less than 0.03 eV/Å, the total stress tensor was reduced to the order of 0.05 GPa, and the maximum ionic displacement was within 0.001 Å. The elements investigated in valence states were O(2s^2^2p^4^), Al(3s^2^3p^1^), Cr(3s^2^3p^6^3d^5^4s^1^), Mn(3d^5^4s^2^), Fe(3d^6^4s^2^), and Co(3d^7^4s^2^). Uspcc pseudo-potentials were used for Mn, Fe, and Co, and usp pseudo-potentials for the rest of the elements. The cell parameters and atomic coordination were fully relaxed during the geometry optimization using a Broyden–Fletcher–Goldfarb–Shanno (BFGS) minimization algorithm. The crystal symmetry was removed by imposing different initial magnetic moments on TM ions so that the electronic ground state could adopt lower symmetry.

## Results and Discussion

**Fig. 1:**
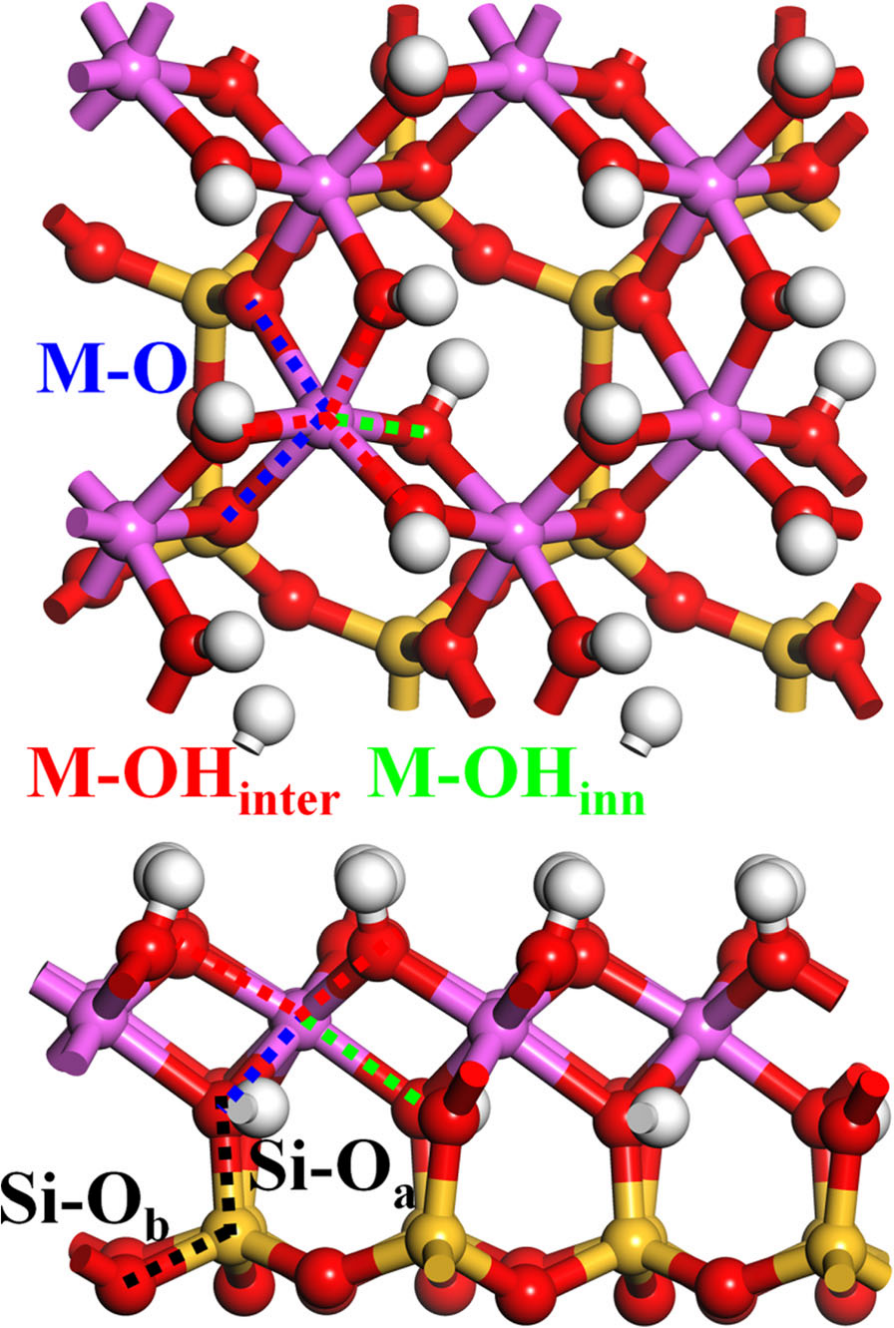
Top (*up*) and side (*down*) views of kaolinite. The Si–O_a_ (*black*), Si–O_b_ (*black*), M–OH_inter_ (*red*), M–OH_inner_ (*green*), and M–O (*blue*) bonds are indicated by *dot lines*

The initial kaolinite structure was taken for our previous work [1]. Figure 1 shows the relaxed 2 × 2 × 1 crystal structure of kaolinite (4 kaolinite units). The kaolinite layer structure, Al_2_Si_2_O_5_(OH)_4_, is composed by an octahedral Al–O sheet and a tetrahedral Si–O sheet, connected by apical O atom (O_a_). The Si–O tetrahedron is constructed by one central Si atom and four surrounding O atom, in which one is the O_a_ atom and the other three are the basal O atoms (O_b_). The Al–O octahedron is constructed by one central Al and six surrounding O, in which two are O_a_ atom and the other four are O atoms (in OH groups) shared with other Al–O octahedron. Besides, these OH groups can be divided into two kinds: the inter-layer OH (OH_inter_) at the surface of the layer structure and the inner OH (OH_inner_) inside the layer structure between the Al sheet and the Si sheet. Hence, there are two kinds of Si–O bonds, Si–O_a_ and Si–O_b_ (black dot line), and three kinds of Al–O bonds, Al–O_inter_ (red dot line), Al–O_inner_ (green dot line), and Al–O (black dot line) in kaolinite bulk structure.

The dispersion energy always plays a major role in the structure stabilization of clay mineral due to the interaction between the layers [21, 43]. Among the several hybrid functionals, PBE-D2 [21], B3LYP [22], B3LYP-D [18], and RPBE-D2 [18, 21], which was used to obtain the experimental lattice structure of kaolinite [44, 45], PBE-D2 functional was found both accurate and less time consuming. The overestimation of PBE functional for bond lengths are overcome by dispersion correction compared to experimental results, as briefly reported previously [1]. In order to distinguish the effect of TM doping on the structure of kaolinite, here, we first revisit the lattice structure and the optimized bond distances between central cations (Si and Al) and oxygen atoms, O_a_, O_b_, and OH_inn_.

**Fig. 2:**
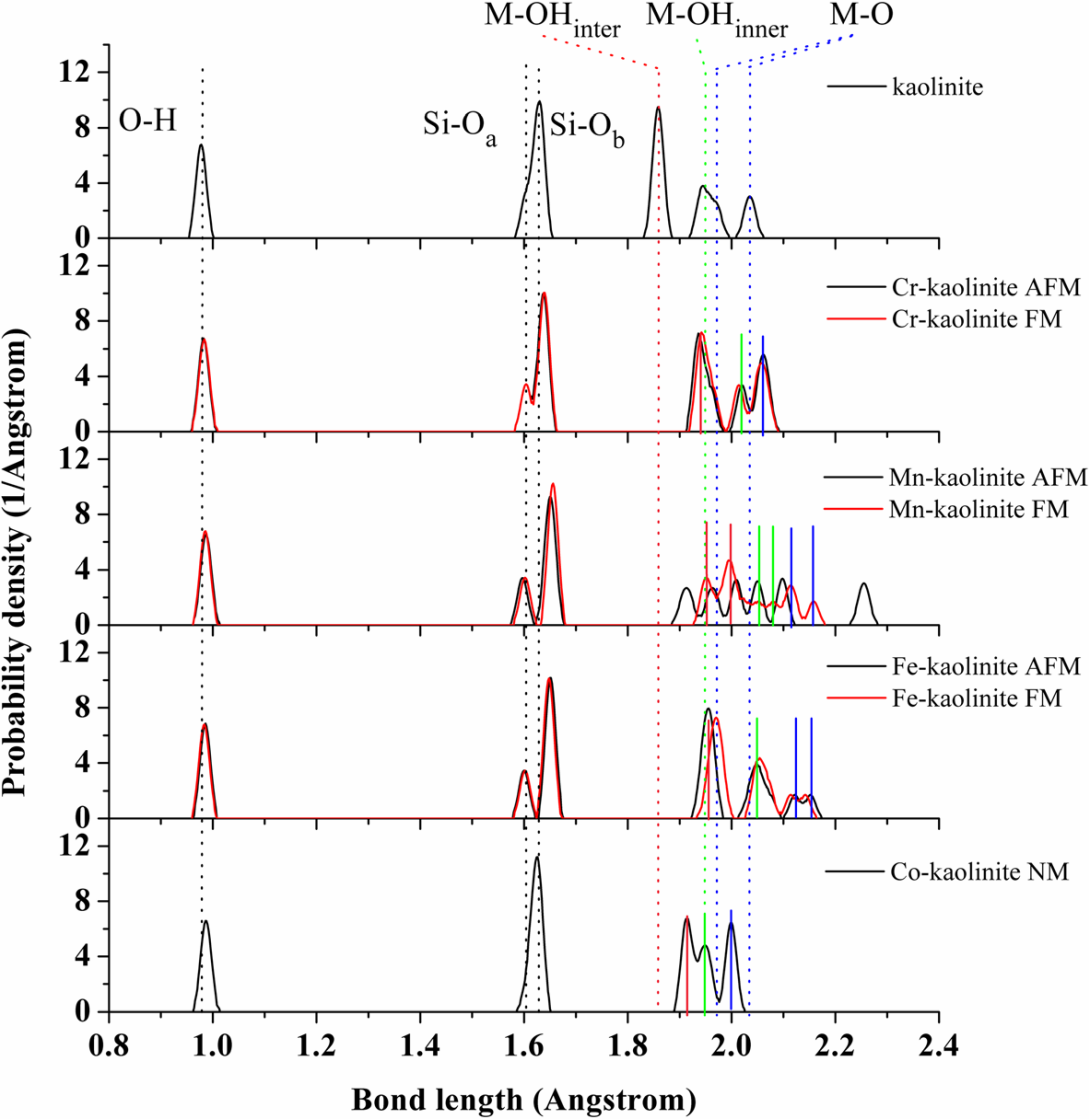
The bond distribution of Cr–, Mn–, Fe–, and Co–kaolinite. Multi-magnetic states are given for each TM–kaolinite. The averaged different types of O–H (*black*), Si–O_a_ (*black*), Si–O_b_ (*black*), M–OH_inter_ (*red*), M–OH_inner_ (*green*), and M–O (*blue*) bonds in kaolinite are indicated by *dot lines*. The M–OH_inter_ (*red*), M–OH_inner_ (*green*), and M–O (*blue*) bonds in Cr–kaolinite (*AFM*), Mn–kaolinite (*FM*), Fe–kaolinite (*AFM*), and Co–kaolinite (*NM*) are indicated by *solid lines*

As shown in Table 1, for kaolinite, the calculated unit cell volume optimized using dispersion-corrected PBE-D2 functional is close to the experimental value, which gives significantly lowered relative error (∼0.4%) compared to PBE functional (∼3.4%). For lattice vectors a and b, the relative error using PBE-D2 (∼0.4%) is much lower than PBE (∼1.1%). And, under dispersion corrections of PBE-D2, the layer distance (vector c) of kaolinite is decreased by 0.17 Å (∼2%). Notably, the lattice angles after dispersion correction are very close to experimental results, especially for α. As for bond length distributions in kaolinite, although PBE-D2 gives little improvement for Si–O_a_, Al–OH_inner_, and Al–O bonds compared with experimental results, a huge improvement is made for Al–OH_inter_ bond at Al–O surface (which is important for surface chemistry) and slight improvement for Si–O_b_ bond at Si–O surface. Notably, for Al–OH_inter_ bond, the dispersion correction from PBE-D2 seems to accurately describe the bonding environment at outmost layer of the Al–O surface, which is strongly influenced by the dispersion force from the Si–O surface of another kaolinite layer that lies above. Another point to mention here is that there are actually two splitted Al–O bonds (Fig. 1, blue dot line) with significantly different bond lengths of about 1.95 and 2.00 Å [45], which shows the lattice distortion of the Al–O octahedron originated from the lattice mismatch between Si–O sheet and Al–O sheet. As a major error in the calculation of kaolinite structure compared to experimental results, these Al–O bonds are overestimated by both PBE and PBE-D2, with similar averaged bond length (Table 1). PBE-D2 gives two Al–O bonds of approximately 1.96 and 2.04 Å, with the second one overestimated by 0.04 Å (Fig. 2, blue dot line).

**Table 1:** Calculated and experimental unit cell parameters (Å) and averaged bond lengths (Å) of kaolinite and the most stable TM–kaolinite structure with AFM, FM, and NM states. The M–O bond represents the Al–O bond for kaolinite or the TM–O bond for TM–kaolinite. The average charge and spin of M atoms are also given

	Kaolinite			Cr–kaolinite		Mn–kaolinite		Fe–kaolinite		Co–kaolinite
	NM			AFM	FM	AFM	FM	AFM	FM	NM
	PBE	PBE-D2	Exp [45]	PBE-D2						
V (Å^3^)	340.99	328.47	329.91	338.22	339.59	349.70	354.59	347.71	348.32	323.96
a (Å)	5.209	5.177	5.154	5.224	5.238	5.242	5.319	5.291	5.282	5.126
b (Å)	9.042	8.981	8.942	9.068	9.092	9.234	9.249	9.183	9.173	8.894
c (Å)	7.483	7.308	7.401	7.374	7.368	7.41	7.44	7.407	7.437	7.314
α (deg)	91.78	91.69	91.69	90.89	90.89	88.28	91.17	91.6	91.46	90.44
β (deg)	104.30	104.69	104.61	104.45	104.55	102.64	104.31	104.87	104.76	103.73
γ (deg)	89.81	89.82	89.82	89.87	89.84	88.76	89.77	89.92	89.88	89.83
Si–O_b_	1.634	1.630	1.620	1.637	1.639	1.650	1.655	1.651	1.648	1.627
Si–O_a_	1.610	1.605	1.610	1.601	1.604	1.596	1.602	1.600	1.602	1.610
M–O	2.004	2.000	1.971	2.061	2.058	2.214	2.117	2.100	2.098	1.999
M–OH_inner_	1.937	1.941	1.921	2.020	2.014	2.050	2.038	2.042	2.048	1.956
M–OH_inter_	1.867	1.859	1.857	1.943	1.948	1.962	1.977	1.954	1.969	1.922
O–H	0.974	0.977		0.983	0.983	0.987	0.986	0.985	0.984	0.987
Charge	1.84	1.84		1.25	1.26	1.21	1.24	1.37	1.37	1.08
Spin	0.00	0.00		3.06	3.08	3.80	3.84	4.02	4.06	0.00

The transition metals (Cr, Mn, Fe, and Co) doped kaolinites were constructed by replacing Al atom with Cr, Mn, Fe, or Co atom. Only the equivalent substitution of Al^3+^ ion with TM^3+^ ion was considered since nonequivalent substitution of TM ions with chemical state other than +3 will cause additional vacancies or impurities for charge balance. From structure point of view, PBE and PBE-D2 functionals of TM–kaolinite give similar structure difference as observed for kaolinite. Considering that PBE-D2 functional describes better for lattice vectors and bond lengths of the two basal surfaces of kaolinite, following discussion on TM–kaolinite, mainly depended on the results obtained by PBE-D2 functional. The lattice parameters, bond length, charge, and spin of TM doped kaolinite and their magnetic states were summarized in Table 1. The energy differences (per TM atom) between AFM and FM states for Cr–kaolinite, Mn–kaolinite, and Fe–kaolinite are 0.022, −0.006, and 0.094 eV, respectively. Since Co–kaolinite structure is only stable at a nonmagnetic state, only NM structure of Co–kaolinite is shown.

The unit cell volumes of TM–kaolinite are expanded compared to kaolinite, with a trend of Mn–kaolinite > Fe–kaolinite >> Cr–kaolinite >> kaolinite > Co–kaolinite. The cell expansions are mainly caused by the longer M–O bonds compared to Al–O bonds, leading to the major expansion in lattice vectors a and b. Meanwhile, the Si–O_b_ bonds at the Si–O sheet are elongated simultaneously, and the crystal lattice angles of α and β are distorted accordingly. The cell volume of Mn–kaolinite with FM state is increased by 1.4% compared to AFM state, while in contrast little influence of magnetic ordering on cell volumes is found for Cr–kaolinite and Fe–kaolinite. The magnetic moments of Cr, Mn, Fe, and Co are close to that in TM doped Al_2_O_3_ [33], while the Mulliken charge are slightly higher which implies stronger reactivity.

The bond length distributions of TM–kaolinite are analyzed in Fig. 2, with different types of Si–O and M–O bonds in TM–kaolinite indicated by solid lines for each doping element. Overall speaking, there is an increase of the bond lengths of M–O and Si–O_b_ after TM doping, and meanwhile there is a reorganization of the bond distribution of the splitted M–O bonds for M–OH_inter_ (red), M–OH_inner_ (green), and M–O (blue) bonds. Notably, the splitted Al–O bonds (blue dot line) disappeared after Cr and Co doping. Furthermore, the bond length distributions are highly dependent on the magnetic ordering for Mn atoms but are only slightly influenced for Cr and Fe atoms.

**Fig. 3:**
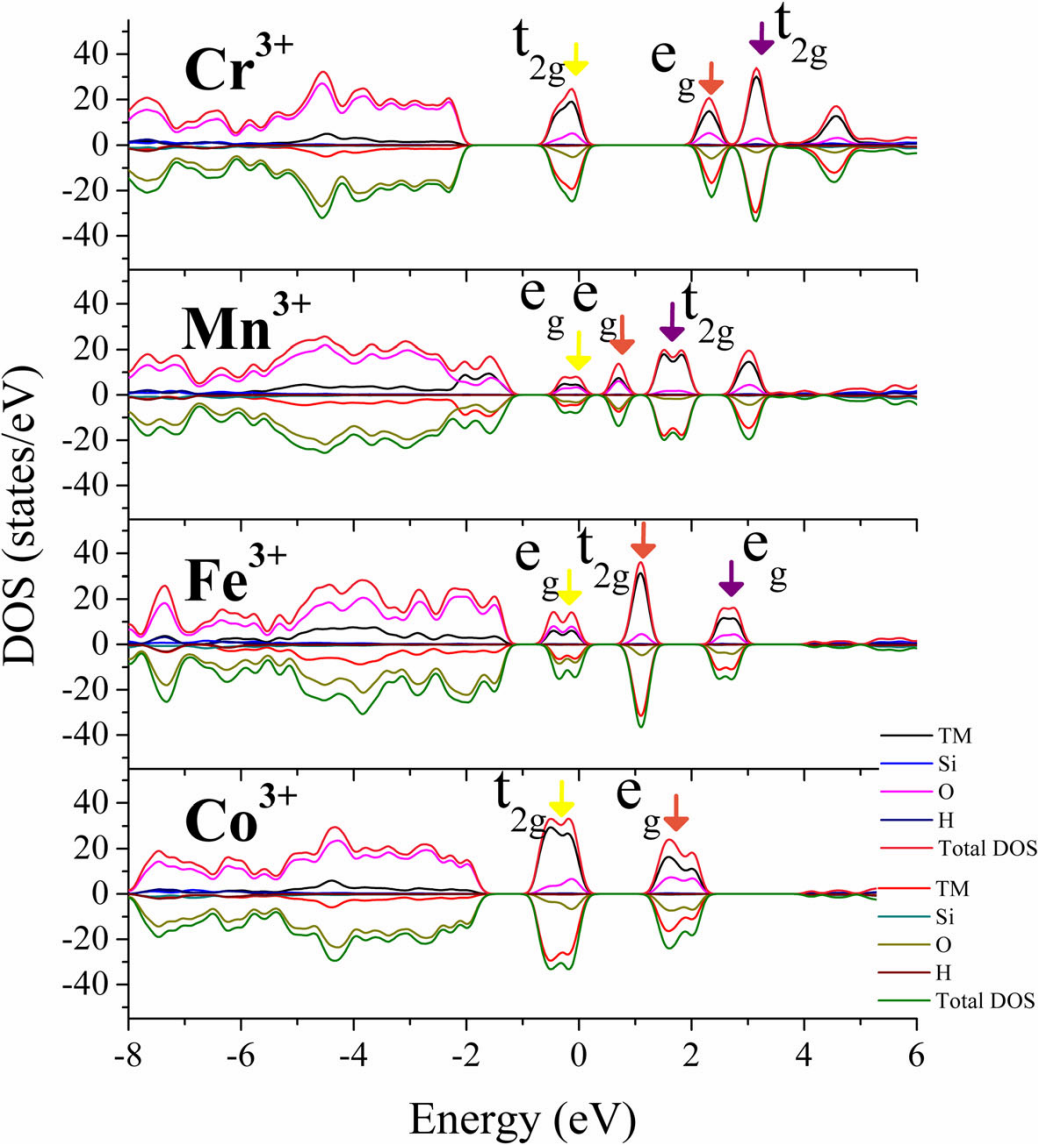
Total density of states (DOS) and atom-projected density of states (PDOS) of the most stable states for TM doped kaolinite are given. The highest occupied 3d orbitals (*yellow*) and the first (*brown*) and second (*purple*) lowest unoccupied 3d orbitals around Fermi level are pointed by *colored arrows*

**Fig. 4:**
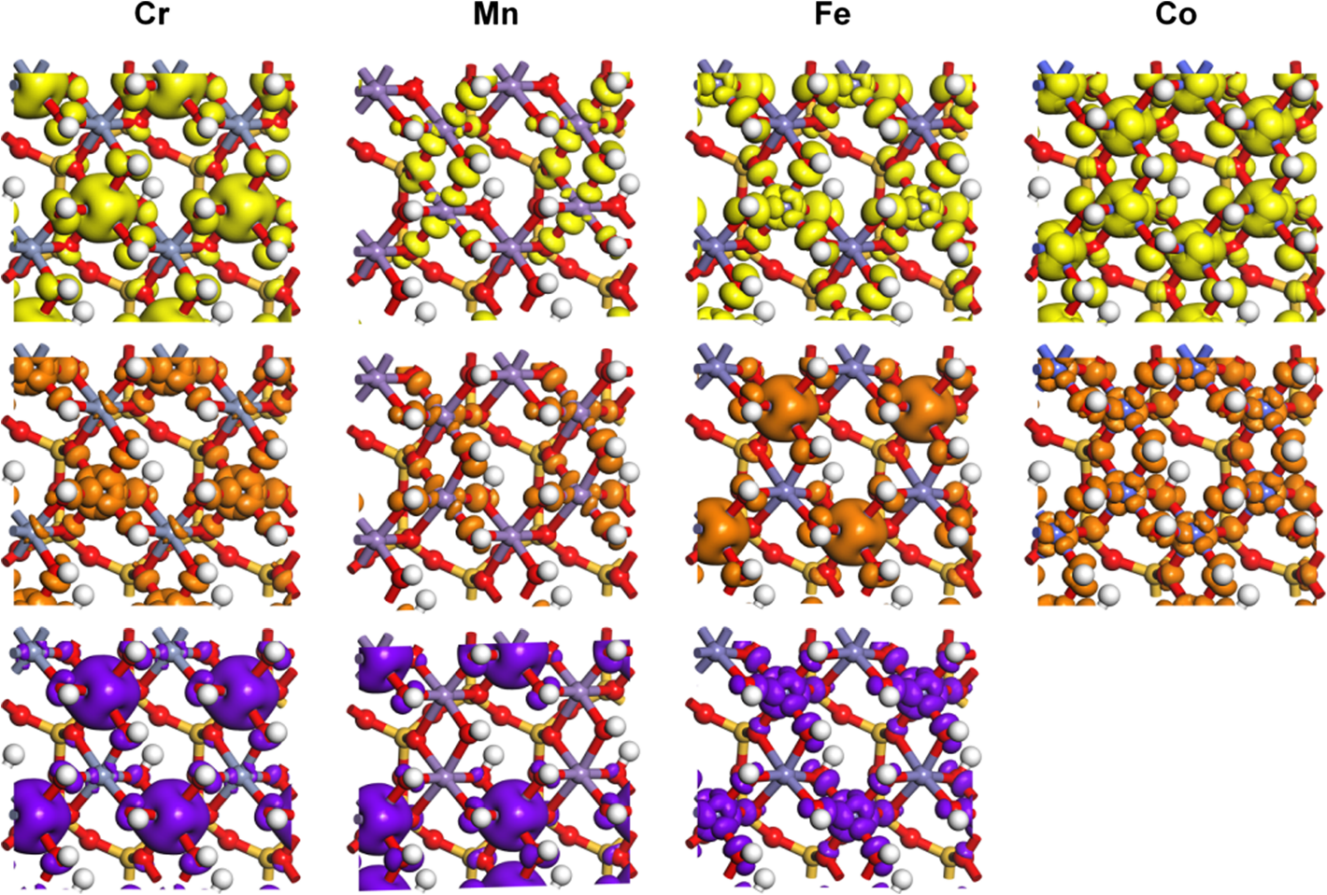
Partial charge density of TM 3d orbitals in TM–kaolinite, corresponding to the states pointed by *arrows* in PDOS results. The isosurface levels are 0.02 e/Å^3^

The PDOS results for Cr^3+^(d3), Mn^3+^(d4), Fe^3+^(d5), and Co^3+^(d6) and the corresponding charge density distributions are shown in Figs. 3 and 4. According to the Jahn–Teller theorem, any degenerate electronic system will spontaneously distort in such a way as to remove the degeneracy [46], which is affected by the surrounding bonding environment [47]. For TM^3+^ doping in octahedral Al site of kaolinite with plenty of hydroxyl groups, the five d-shell orbitals of TM^3+^ will split into a triplet t_2g_ state and a doublet e_g_ state under Oh symmetry. The electrons in the triplet state are localized in the middle region between the ligands and further hybridized with the nearest O states. Those in the doublet state point directly at the ligands and thus lie higher in energy than the t_2g_ electrons. Generally, the presence of electrons in the e_g_ orbitals tends to destabilize the octahedral bonding, and the degeneracy is removed by lengthening the bonds opposite the filled orbital and shortening the bonds opposite the empty orbital. The d–d transition of TM^3+^(Oh) species is always from the occupied t_2g_ orbitals (dxy, dyz, and dzx) to unoccupied e_g_ orbital (d_x2-y2_ or d_z2_, depending on their occupancy). The orbital splitting between e_g_ orbitals and t_2g_ orbitals of Cr^3+^(d^3^), Mn^3+^(d^4^), Fe^3+^ (d^5^), and Co^3+^ (d^6^) in TM–kaolinite is similar with that in Al_2_O_3_ and TiO_2_ [33, 48, 49], but the splitting energies between 3d orbitals are slightly larger than in their own oxides (Fig. 3), possibly due to the hybridization with the surrounding hydroxyl groups.

The difference of splitting energies between FM and AFM states of Mn–kaolinite is small, and the distributions of density of states are similar except the spin directions are different. Hence, for simplicity, only the results for AFM state are shown. For the high-spin Mn^3+^(d^4^) ion in Mn–kaolinite with AFM state, only one of the two e_g_ orbitals is occupied at the valence band maximum (VBM) (Fig. 3, yellow arrow). The occupation of d_z2_ orbital which is lower in energy gives a strong repulsion on the bonding electrons of the two ligands along the *z* axis and elongates the M–O bonding in that direction. This effect is the well-known Jahn–Teller effect. The states at the bottom of the conduction band minimum (CBM) are composed by the lowest unoccupied d_z2_ orbital (brown arrow) and the higher d_x2y2_ orbital (purple arrow) of Mn^3+^(d^4^). For Cr^3+^(d^3^), Fe^3+^ (d^5^), and Co^3+^ (d^6^) doped case, where the t_2g_ and e_g_ orbitals are occupied evenly, the influence of Jahn–Teller distortion effect is small, which only caused slight deviation of the M–O bonds in TM–kaolinite (Fig. 2). Such modification of structure and electronic properties by TM doping might improve the application of kaolin in the field of catalysis [50, 51], CO capture [52, 53], drug loading [54], and energy storage [55–57]. And, it can also be applied to other minerals, such as montmorillonite [50, 58], perlite [55], and talc [59] to alter their electronic properties.

## Conclusions

The influence of transition metal (Cr, Mn, Fe, and Co) doping on geometric structure and electronic structure of kaolinite nanoclay are investigated by DFT calculations. The crystal volume, lattice parameters, bond length, charge and spin, and possible magnetic states are calculated and studied. The Cr^3+^ and Fe^3+^ dopants show more stable under AFM state, Mn^3+^ prefer FM state, and Co^3+^ dopants prefer NM state. The transition metal doping induces lattice volume expansion and some reorganization of the M–O bond distributions. Meanwhile, the TM dopants introduce some 3d states with larger splitting energies in the band gap of kaolinite.

## Abbreviations

AFM: Antiferromagnetic
BFGS: Broyden–Fletcher–Goldfarb–Shanno
CASTEP: Cambridge Sequential Total Energy Package
CBM: Conduction band minimum
DFT: Density functional theory
DFT-D2: Dispersion-corrected density functional theory
FM: Ferromagnetic
GGA: Generalized gradient approximation
NM: Nonmagnetic
PBE: Perdew, Burke, and Ernzerhof
SCF: Self-consistent field
TM: Transition metal
VBM: Valence band maximum
